# *Palaeoamyda messeliana* nov. comb. (Testudines, Pan-Trionychidae) from the Eocene Messel Pit and Geiseltal localities, Germany, taxonomic and phylogenetic insights

**DOI:** 10.7717/peerj.2647

**Published:** 2016-10-27

**Authors:** Edwin Cadena

**Affiliations:** School of Geological Sciences and Engineering, Yachay Tech, San Miguel de Urcuquí, Imbabura, Ecuador

**Keywords:** Trionychidae, Geiseltal, Eocene, Germany, Messel Pit

## Abstract

**Background:**

Abundant pan-trionychid (soft-shell) turtles specimens have been found in Eocene sequences of central Europe, particularly from two localities in Germany, the Messel Pit (a UNESCO World Natural Heritage Site) and Geiseltal, traditionally attributed to *Trionyx messelianus* or *Rafetoides austriacus*. Over the last two decades new specimens of this taxon from these two localities have been discovered and fully prepared. However, they have remained unstudied, as well as their phylogenetic position inside Pan-Trionychidae is unknown.

**Results:**

Five new specimens of *Palaeoamyda messeliana* nov. comb. from Messel Pit and Geiseltal localities are fully described here. A revised diagnosis for the species is also presented here, together with its inclusion in a phylogenetic analysis of Pan-Trionychidae that shows that this species is sister to the extant *Amyda cartilaginea*, one of the most abundant pan-trionychid (soft-shell) turtles from Asia, both members of the clade Chitrini. The specimens described in here are among the best and most complete fossil pan-trionychid skeletons so far known.

## Introduction

Trionychia constitutes one of the six major lineages of turtles, more recently considered as the sister group to all other cryptodires, or hidden-necked turtles (Crawford et al., 2015). With a fossil record that extends from the early Cretaceous to present, Pan-Trionychidae is represented by at least 300 fossil and extant species, and has a geographical distribution that has principally been restricted to the northern continents (Laurasia) (Meylan, 1987; Danilov & Vitek, 2013; Joyce et al., 2013; Crawford et al., 2015; Vitek & Joyce, 2015). Trionychians are represented by two major clades (Pan-*Carettochelys* and Pan-Trionychidae) (Joyce, 2014; Vitek & Joyce, 2015). Pan-Trionychidae (pan-trionychids) includes most of the iconic soft-shell turtles.

**Figure 1:**
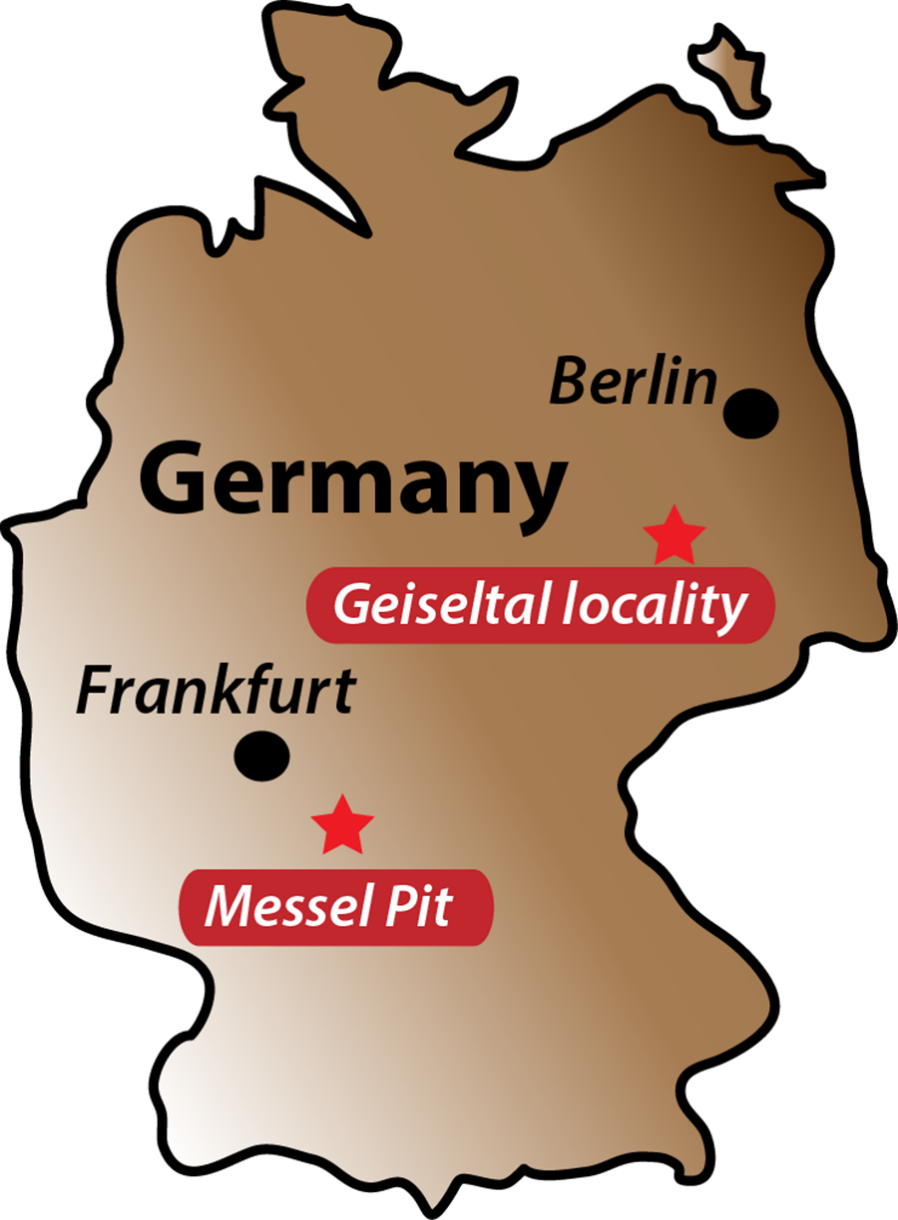
Fossil localities. Map of Germany showing the location of Messel Pit and Geiseltal locality.

Fossils of European Paleogene pan-trionychids have been traditionally grouped in the genus *Trionyx* Geoffroy (1809), (Lapparent de Broin, 2001; Danilov et al., 2011) and in a most recent erected genus: *Rafetoides* Karl, 1998. Paleogene European pan-trionychids show a widespread record in the continent, including the early Eocene of Spain, France, Belgium, Italy, Austria, Germany, Hungary, Romania, England, and Slovenia up to the lower Rupelian, a complete and detail review of the fossil record was recently presented in Georgalis (in press). Two fossil localities in Germany (Fig. 1) preserve some of the most complete Cenozoic European pan-trionychids: the middle Eocene (early Lutetian, MP11, ∼48 Ma) (Lenz et al., 2015) Messel Pit, near Darmstadt, and Geiseltal, Saxony-Anhalt, from the uppermost middle Eocene ∼45 Ma (Haubold & Hellmund, 1998). Fossil pan-trionychids from these both localities have been reported or briefly described by Lapparent de Broin (2001), Karl (1998), Karl (1999), and Karl & Müller (2008). However none of these studies have included them in phylogenetic analyses, leaving their relationships inside Pan-Trionychidae poorly unknown. Early works that described the pan-trionychid from Messel Pit defined this taxon as *Trionyx messelianus* Reinach, 1900, later, two additional varieties were considered: *T. messelianus* var. *lepsiusi* Harassowitz, 1919, and *T. messelianus* var. *kochi* Hummel, 1927, this last work defined a new combination for the species: *T*. (*Amyda*) *messelianus*. More recently Karl (1997) and Karl (1998) considered *T*.(*Amyda*) *messelianus* as junior synonym of the Croatian taxon *T. austriacus* Peters, 1859, having affinities with the English taxon *T. henrici* Owen & Bell, 1849 and united all of them in a new genus called *Rafetoides.* However, the use of the *Rafetoides* genus for the Messel Pit pan-trionychids has been rejected recently in the review on the Old World pan-trionychids (Georgalis , in press), indicating that *Trionyx austriacus* is a nomen dubium and using the name *T. messelianus* for the Messel forms. As it is discussed herein, the generic assignment of “*Trionyx*” *messelianus* appears to be problematic, making the necessity of formulate a new genus name for this species, something that is done here following the rules of the Code of the International Commission on Zoological Nomenclature (ICZN, 1999).

Over the last two decades, new specimens of pan-trionychid turtles from Messel and Geiseltal have been collected and fully prepared, but they have remained undescribed. Furthermore, some older specimens, previously kept in a private collection and inaccessible to study, have recently been accessioned at the WDC, as is also the case for the Geiseltal specimen described here. In this paper, I describe a total of four new specimens from Messel and one from Geiseltal. All these specimens pertain to the former *Trionyx messelianus* (with a new genus name defined herein), which I include for first time in a phylogenetic analysis of Pan-Trionychidae. This work also constitutes a contribution to the understanding of the turtle fauna from Messel pit, which recently has been involved in two main studies, one describing the carettochelyid turtle species *Allaeochelys crassesculpta* including specimens that died while they were copulating (Joyce et al., 2012) and the redescription and phylogeny of the podocnemidid turtle *Neochelys franzeni* (Cadena, 2015).

## Material and Methods

### Material

The four new specimens from Messel pit are housed at the Senckenberg Museum collections in Frankfurt, Germany (numbers SMF ME 1211 and 1003), and the Wyoming Dinosaur Center (numbers WDC-C-MG-310 and -311). The specimen from Geiseltal is housed at the Wyoming Dinosaur Center (number WDC-PBP-2015-DE001). For comparisons, complete skeletons of extant species were directly examined including: *Amyda cartilaginea* Boddaert, 1770 specimen NHMW 32232, *Apalone spinifera* Lesueur, 1827 specimen NHMW 1876, *Rafetus euphraticus* Daudin, 1801 specimen NHMW 1856, and the computer tomography skull material available at Digimorph.org for *Apalone mutica* Lesueur, 1827 specimen PCHP 2746 and *Trionyx triunguis* Forskål, 1775 specimen PCHP 4559.

### Phylogenetic analysis

For the phylogenetic analysis, the new taxon was added to the character-taxon matrix of Joyce & Lyson (2011), for a total of 27 taxa and 83 morphological characters (Supplemental Information 1, Nexus file). Taxon names were updated following Turtle Taxonomy Working Grop (2014). The analysis was run using PAUP v4.0a149 (Swofford, 2002), all characters were considered unordered and equal weighted, multistate taxa interpreted as polymorphism, gaps treated as “missing.” A heuristic search was performed using 10,000 replicates and TBR branch-swapping. A second analysis was run using a topological constraint based on molecular trees of Engstrom, Shaffer & Mccord (2004), as was also done by Joyce & Lyson (2010, Fig. 2), enforcing and keeping all the trees compatible with the constraint. Bremer decay indices were obtained.

### Nomenclatural act

The electronic version of this article in Portable Document Format (PDF) will represent a published work according to the International Commission on Zoological Nomenclature (ICZN), and hence the name contained in the electronic version is efectively published under that Code from the electronic edition alone. This published work and the nomenclatural acts it contains have been registered in ZooBank, the online registration system for the ICZN. The ZooBank LSIDs (Life Science Identifiers) can be resolved and the associated information viewed through any standard web browser by appending the LSID to the prefix “http://zoobank.org/”. The LSID for this publication is: (urn:lsid:zoobank.org:pub:A662842B-28A5-4A17-AC91-A9EDE8A75E79). The online version of this work is archived and available from the following digital repositories: PeerJ, PubMed Central and CLOCKSS.

## Systematic Paleontology

**Table:** 

**TESTUDINES** Batsch, 1788
**CRYPTODIRA** Cope, 1868
**PAN-TRIONYCHIDAE** Sensu Joyce, Parham & Gauthier, 2004
***Palaeoamyda* nov. gen.**

**Etymology**

A fossil version of the extant genus *Amyda*, based on the important similarities between these two genera.

**Type species**

*Palaeoamyda* (orig. *Trionyx*) *messeliana* nov. comb. (Reinach, 1900).

**Included species**

*Palaeoamyda messeliana* nov. comb.

**Diagnosis**

Same as for the type species, *Palaeoamyda messeliana* nov. comb.

***Palaeoamyda messeliana* nov. comb.** (Reinach, 1900)

Figs. 2–9

**Figure 2:**
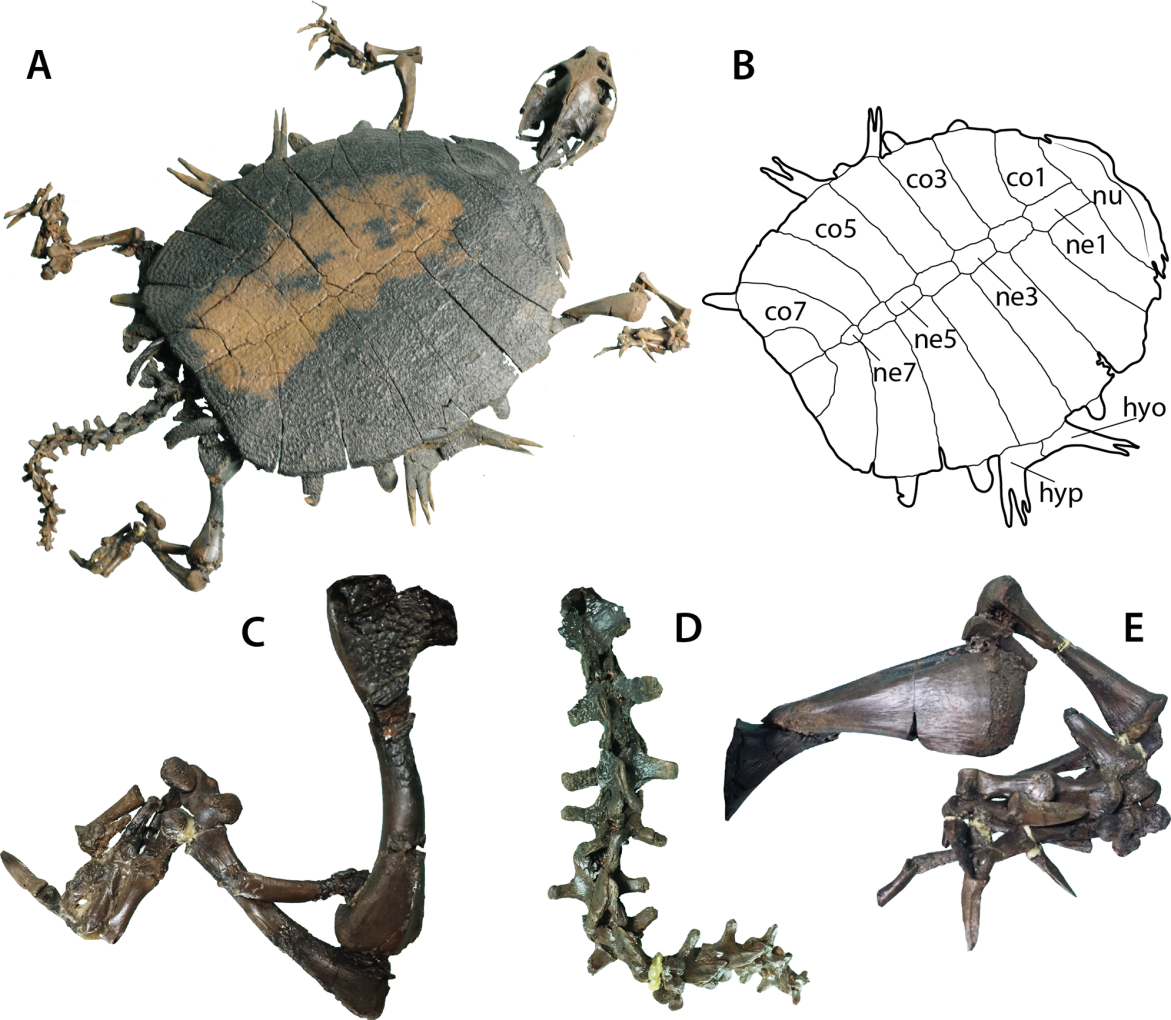
*Palaeoamyda messeliana* SMF ME 1211. (A) articulated skeleton in dorsolateral view. (B) outline of the carapace and lateral plastral elements in dorsolateral view. (C) close-up of the right hindlimb. (D) close-up of the tail. (E) close-up of the right forelimb. Abbreviations: co, costal bone; hyo, hyoplastron; hyp, hypoplastron; ne, neural bone; nu, nuchal bone.

**Figure 3:**
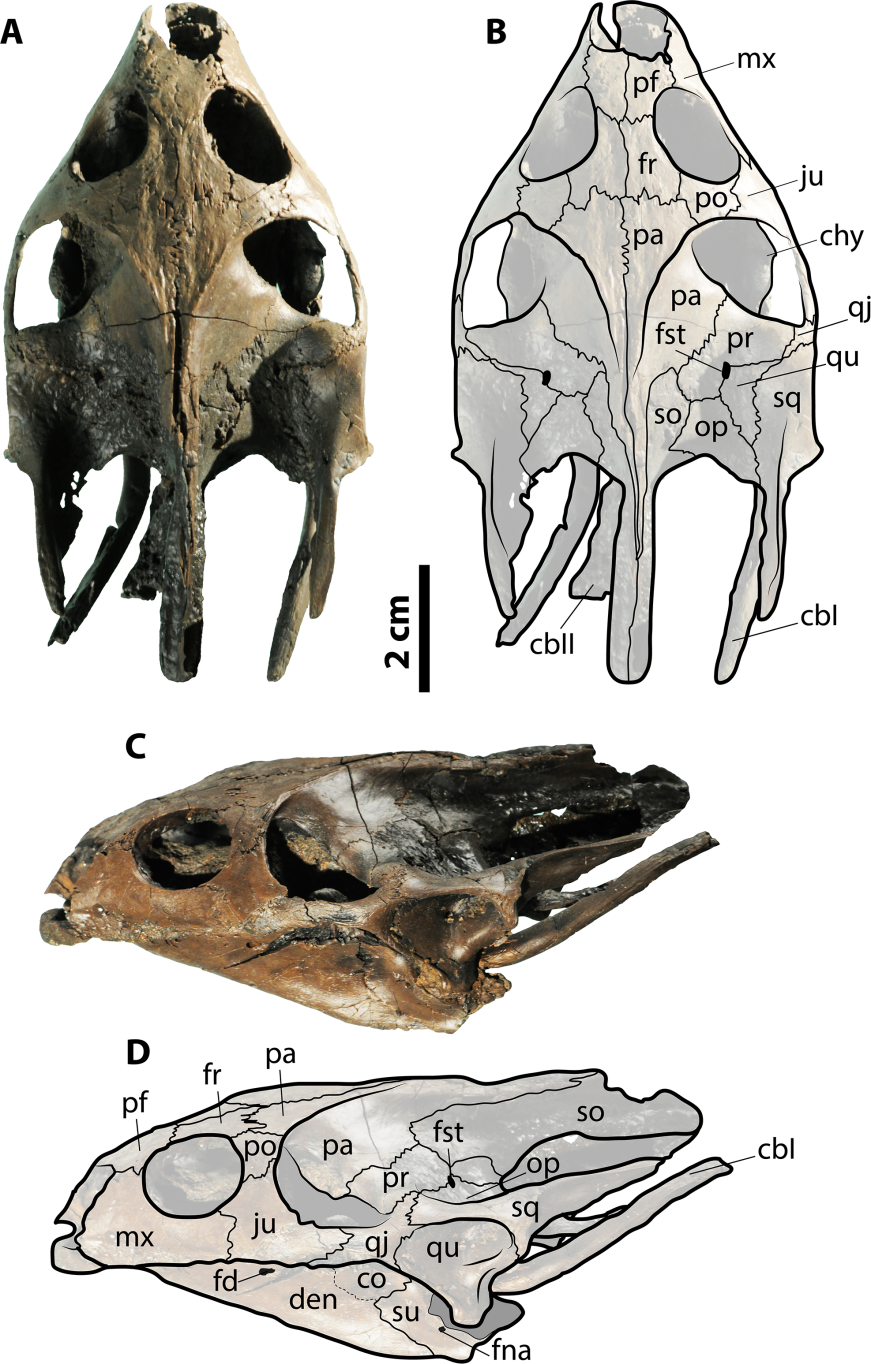
Skull of *Palaeoamyda messeliana* SMF ME 1211. (A–B) skull in dorsal view. (C–D) skull in left lateral view. Abbreviations: cbI, cornu branchiale I; cbII, cornu branchiale II; chy, corpus hyoidis; co, coronoid; den, dentary; fd, foramen dentofaciale majus; fna, foramen nervi auri-culotemporalis; fr, frontal; fst, foramen stapediotemporale; ju, jugal; mx, maxilla; op, opisthotic; p, pa, parietal; pf, prefrontal; po, postorbital; pr, prootic; qj, quadratojugal; qu, quadrate; sq, squamosal; so, supraoccipital; su, surangular. Gray filled areas represent rock matrix.

**Figure 4:**
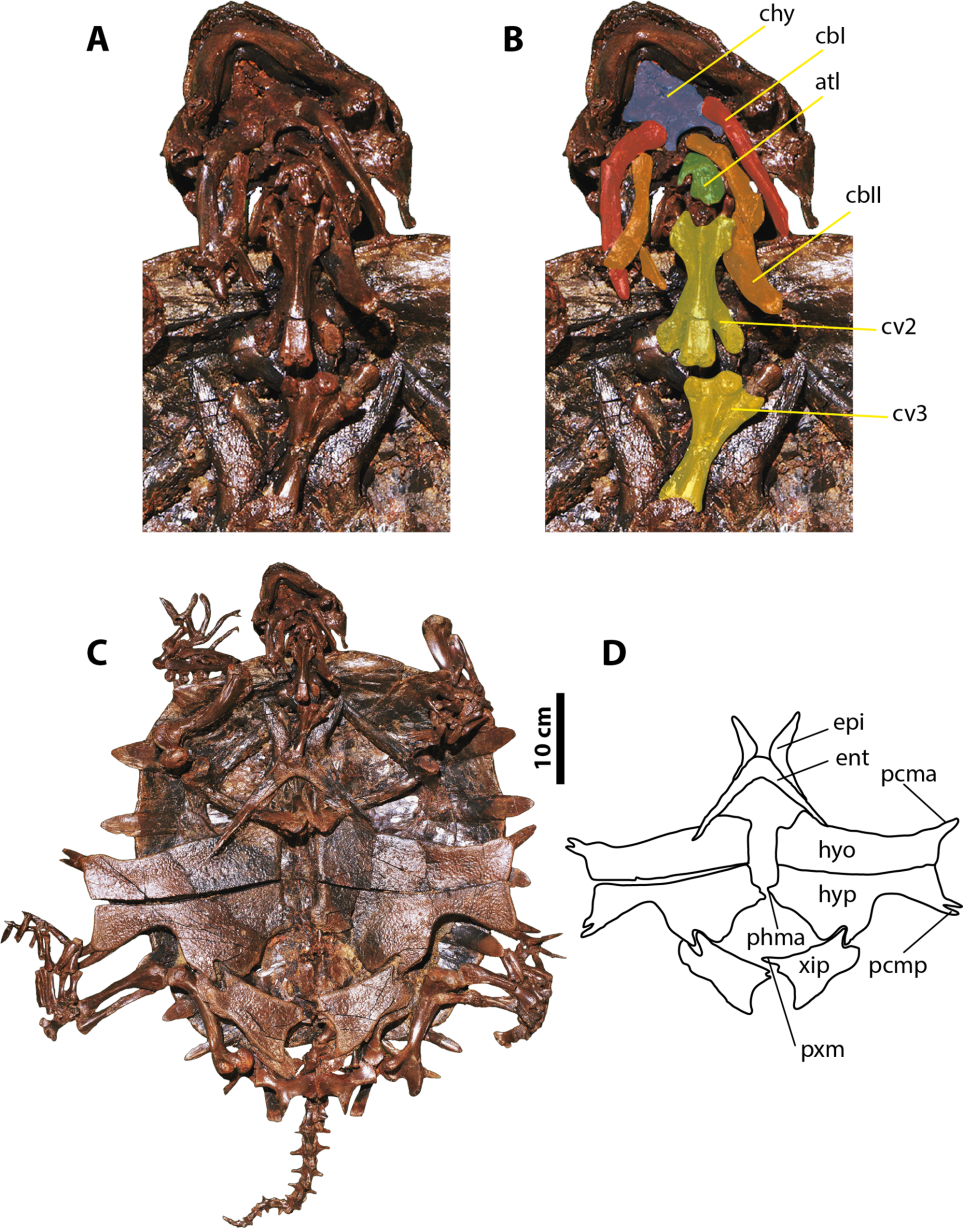
*Palaeoamyda messeliana* SMF ME 1003. (A–B) skull in posteroventral view. (C) complete articulated skeleton in ventral view. (D) outline of the plastron in ventral view. Abbreviations: atl, atlas; cbI, cornu branchiale I; cbII, cornu branchiale II; chy, corpus hyoidis; cv, cervical vertebra; ent, entoplastron; epi, epiplastron; hyo, hyoplastron; hyp, hypoplastron; pcma, processus cardinus masculi anterior; pcmp, processus cardinus masculi posterior, phma, processus hypoplastralis mediales anterior; pxm, processus xiphiplastrales media; xip, xiphiplastron.

**Figure 5:**
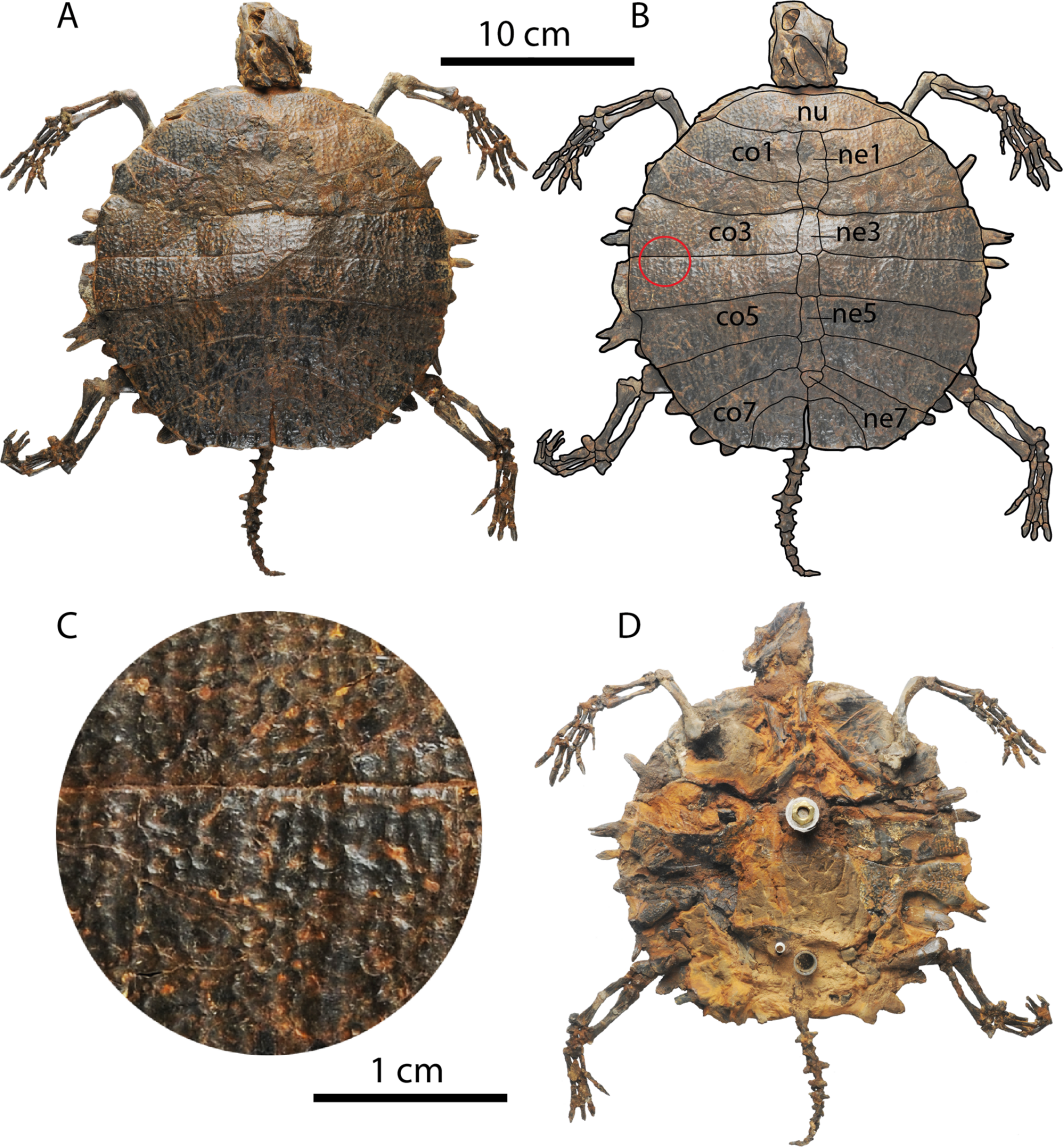
*Palaeoamyda messeliana* WDC-C-MG-310. (A–B) articulated skeleton in dorsal view. (C) close-up showing the sculpturing pattern of the carapace, from red circle in B (1cm scale bar). (D) articulated skeleton in ventral view. Abbreviations: co, costal bone; ne, neural bone; nu, nuchal bone.

**Figure 6:**
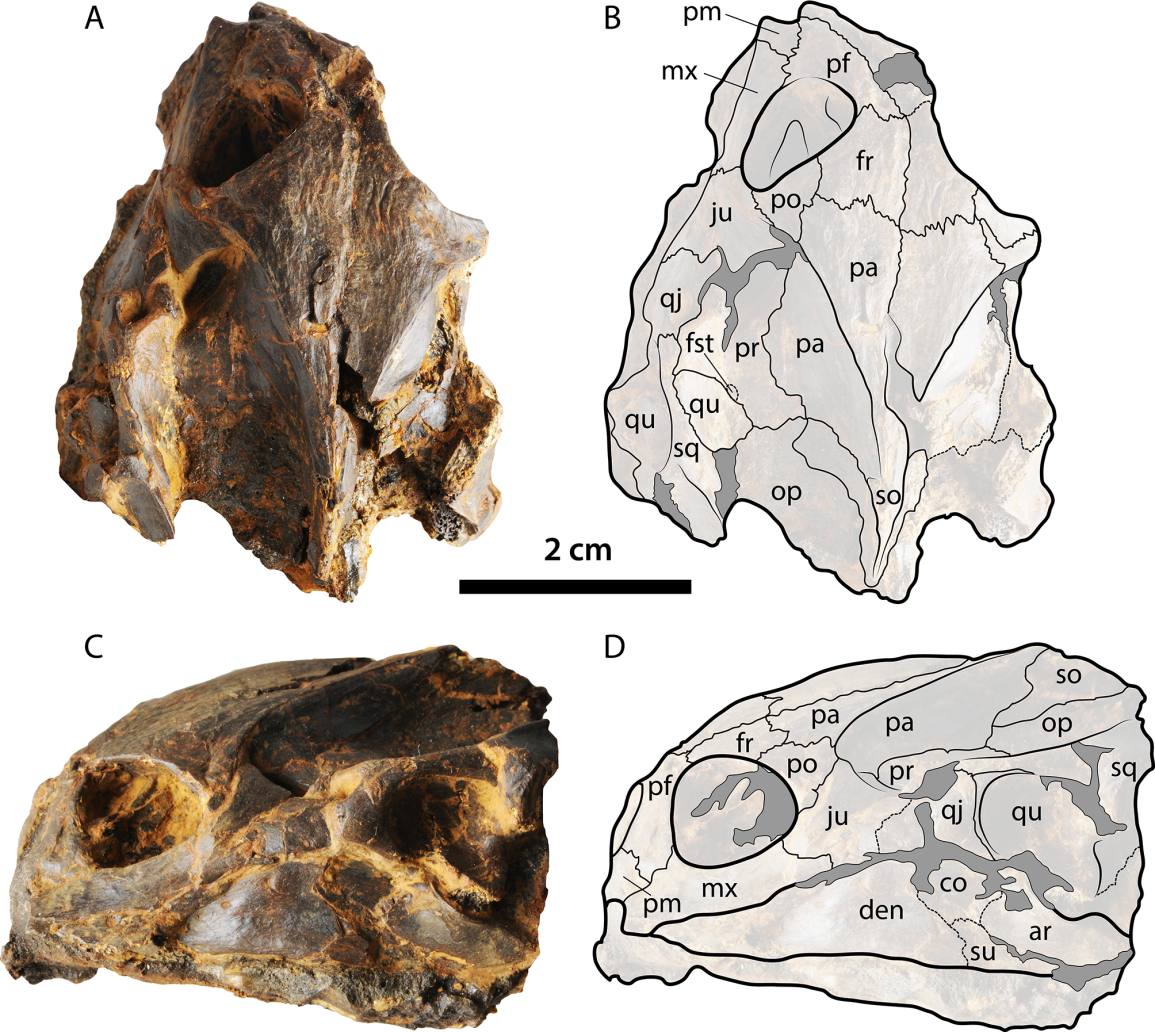
Skull of *Palaeoamyda messeliana* WDC-C-MG-310. (A–B) skull in dorsal view. (C–D) skull in left lateral view. See Fig. 3 for abbreviations. Additional abbreviations: ar, articular; pm, premaxilla.

**Figure 7:**
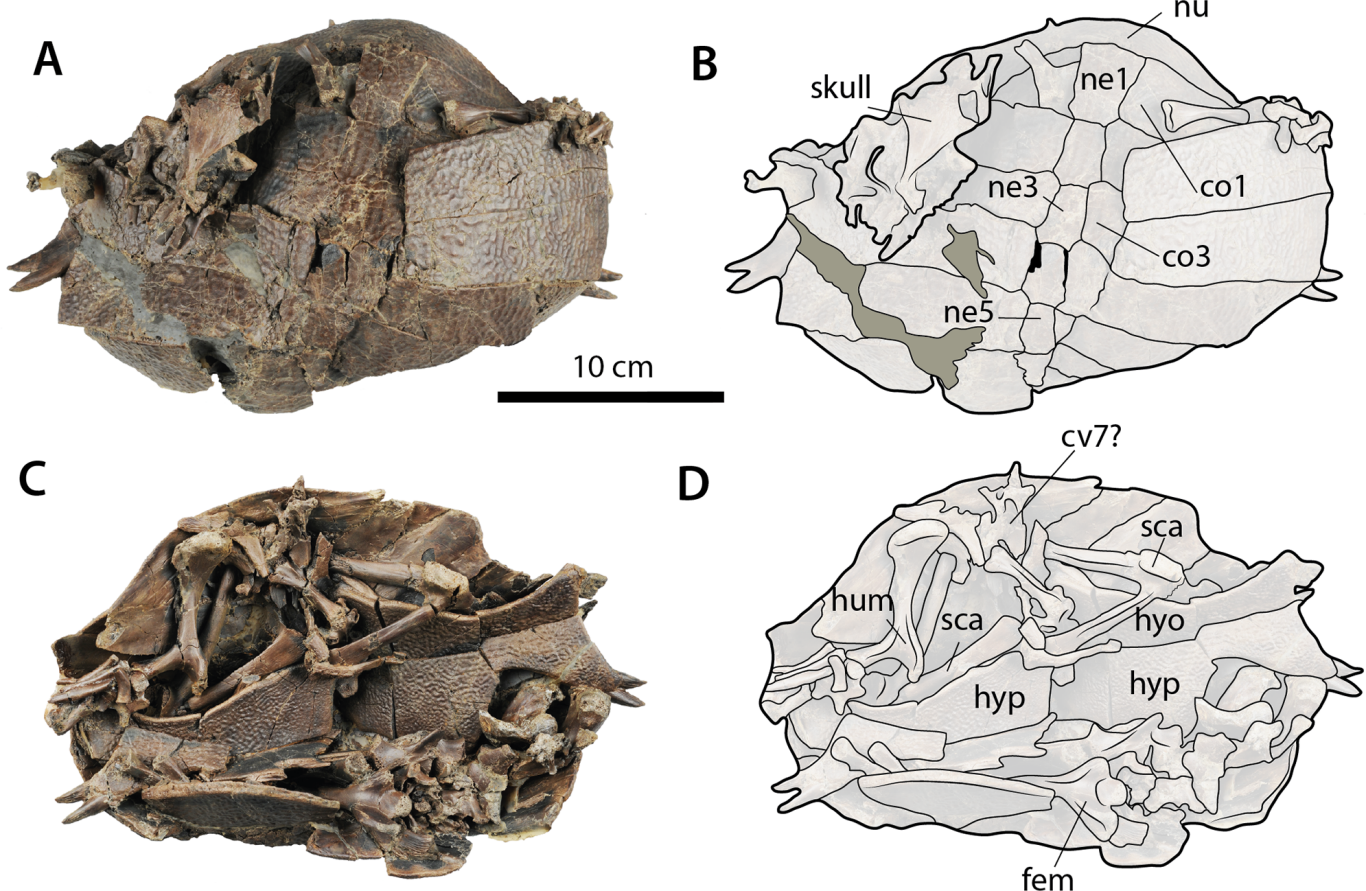
*Palaeoamyda messeliana* WDC-C-MG-311. (A–B) carapace and skull in dorsal view. (C–D) disarticulated skeleton in ventral view. Abbreviations: co, costal bone; cv, cervical vertebra; fem, femur; hum, humerus; hyo, hyoplastron; hyp, hypoplastron; ne, neural bone; nu, nuchal bone, sca, scapula-acromion. Gray filled areas represent rock matrix.

**Figure 8:**
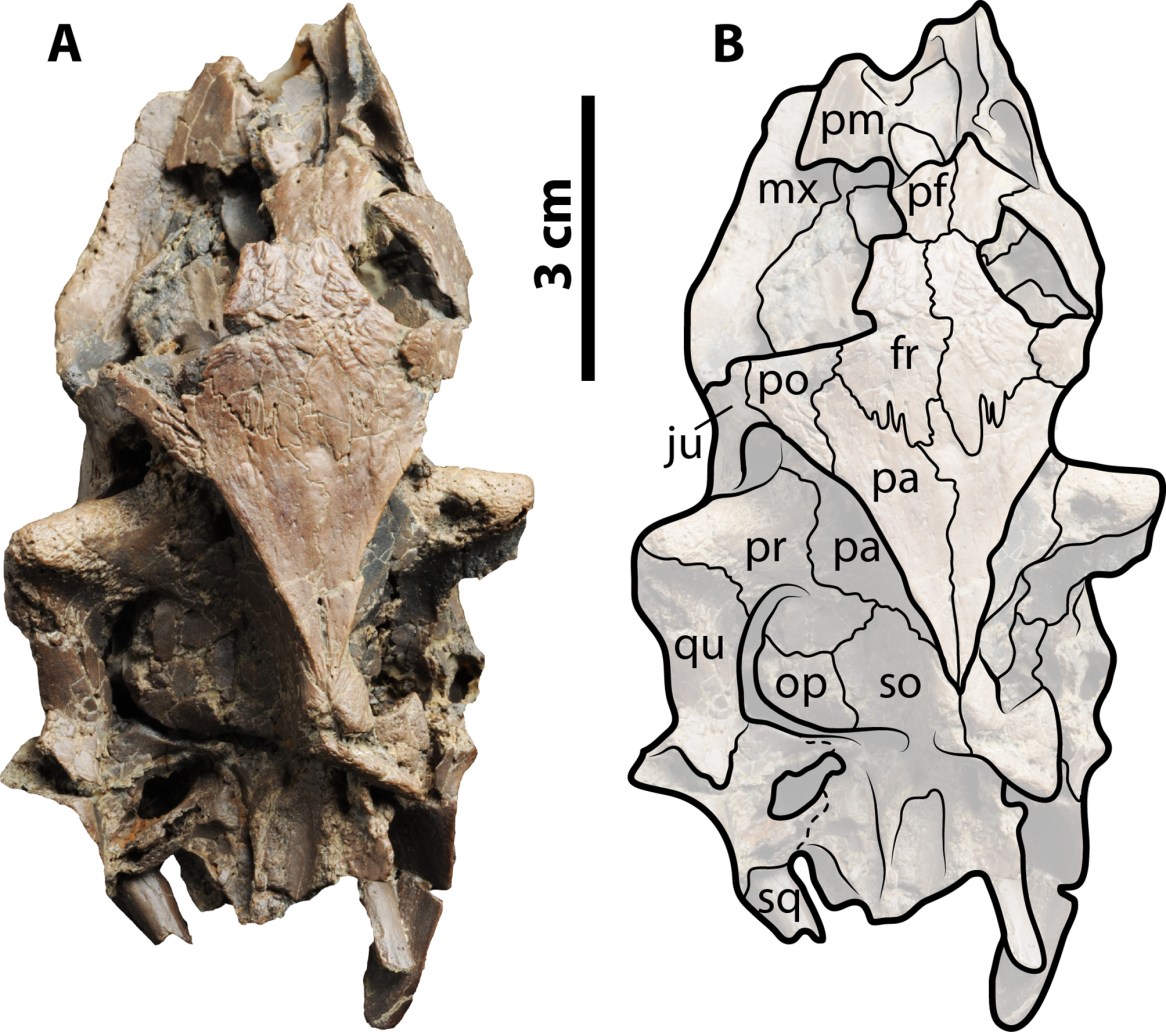
Skull of *Palaeoamyda messeliana* WDC-C-MG-311. (A–B) skull in dorsal view. See Fig. 3 for abbreviations.

**Figure 9:**
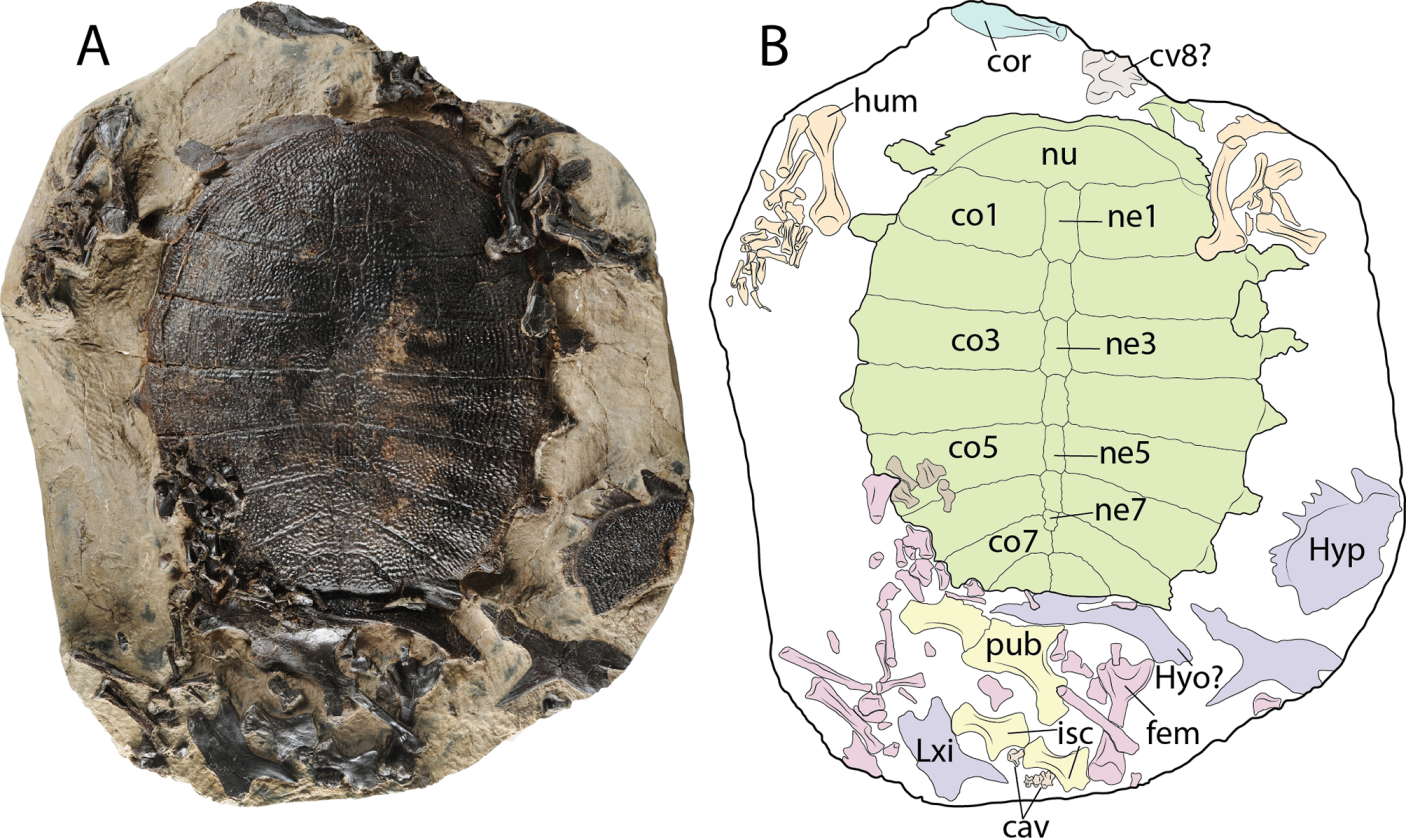
*Palaeoamyda messeliana* WDC-PBD-2015-DE001. (A–B) carapace in dorsal view and some postcranial elements. (C–D) disarticulated skeleton in ventral view. Abbreviations: cav, caudal vertebra; co, costal bone; cor, coracoid; cv, cervical vertebra; fem, femur; hum, humerus; hyo, hyoplastron; hyp, hypoplastron; isc, ischium; ne, neural bone; nu, nuchal bone, pub, pubis; sca, scapula-acromion. Carapace (green), plastron (purple), forelimbs (organge), hindlimbs (pink), pelvic girdle (yellow) cervicals and caudals (gray), coracoid (blue).

**Taxonomic history**

*Trionyx messelianus* Reinach, 1900

*Trionyx messelianus* var. *lepsiusi* Harassowitz, 1919

*Trionyx* (*Amyda*) *messelianus* var. *kochi* Hummel, 1927 (new combination)

*Rafetoides austriacus* Karl, 1997 (junior synonym).

**Type specimen**

SMF ME 106, representing a nearly complete carapace and partial plastron.

**Referred specimens described in this work**

SMF ME 1211, nearly complete skeleton (Figs. 2 and 3); SMF ME 1003 nearly complete skeleton (Fig. 4); WDC-C-MG-311, nearly complete skeleton (Figs. 5 and 6); WDC-C-MG-310, disarticulated shell but with almost all bones preserved in a “cradle” like specimen, skull and some limb bones (Figs. 7 and 8); and WDC-PBD-2015-DE001, complete carapace and some portions of the plastron, limb bones, pelvic-pectoral girdles, and a cervical and some caudal vertebrae (Fig. 9).

**Localities and horizons**

SMF ME specimens were collected in the Messel Pit, near Darmstadt, Germany, middle Eocene (early Lutetian, MP11, ∼47 Ma) (Lenz et al., 2015). WDC specimen was collected in Geiseltal locality, Saxony-Anhalt region, Germany. The age for this locality is middle Eocene Haubold & Hellmund (1998). However, not specific information about the horizon or any other details of the collection are known.

**Revised diagnosis**

*Palaeoamyda messeliana* nov. comb., shares the following synapomorphies with Pan-Trionychidae: contribution of the jugal to the upper temporal emargination, loss of a contribution of the fused premaxilla to the external nares, absence of carapacial and plastral scutes, the absence of pygal bones, sculpturing pattern that covers all metaplastic portions of the shell bones, V-shape entoplastron. *Palaeoamyda messeliana* nov. comb. shares with Trionychinae the following synapomorphies: a nuchal bone at least three times wider than long, complete absence of peripheral bones, neural series always containing at least one reversal of neural orientation, and a short bridge. Inside Trionychinae (sensu Meylan, 1987), *Palaeoamyda messeliana* nov. comb. resembles members of Chitrini in particular *Amyda cartilaginea* by: having a hypoplastron having only one processus hypoplastrales medialis posterior, a pair of processus cardinus masculi anterior, and a very short processus hypoplastralis medialis anterior, a narrow and well developed metischial process of ischium, a very advanced temporal emargination reducing the area of exposure of parietals at the roof top of the skull. Differs from other chitrinins by the following plesiomorphies: costals 7 having a medial contact between each other for half of more of their total medial margin, a shallow medial notch at the anterior margin of nuchal, seven neurals, with the neural reversal of orientation at neural 6. Potential autapomorphies of *Palaeoamyda messeliana* nov. comb., suggested by Karl (1998) brachial horn II with several ossifications, triturating surface completely flat or with solid symphyseal notch, entoplastron having acute angle and being straight at symphysis.

**Remarks**

Comments on other diagnostic characters included by Karl (1998) for *Palaeoamyda messeliana* nov. comb: (1) eight costals: this character is highly variable among pan-trionychids, eight costals being the most common condition for almost all groups, extant and extinct. (2) ischial process (metischial process) well developed: this a character commonly shared by members of the whole Chitrini clade, and not just *P. messeliana*. (3) Preneural lacking permanent postnuchal fontanella: the degree of ossification is high in chitrinins, which lack a fontanelle and instead of a preneural have a long neural 1.

How it is discussed and supported in this study, “*Trionyx*” *messelianus* is not phylogenetically related or morphologically similar with *Trionyx* genus, but instead it is found closer and more similar to the extant genus *Amyda*, reason why I define a new genus name (*Palaeoamyda*) for this taxon, keeping its original valid species name (*messelianus*). However, taking into account that the gender of the new genus name is feminine, the epithet for the species should be also feminine and that is why should be “*messeliana*” instead of “*messelianus*,” resulting in the new combination *Palaeoamyda messeliana.*

## Descriptions

### SMF ME 1211

Nearly complete articulated skeleton (56 cm maximum length and 42 cm maximum width as preserved), including the articulated skull and lower jaw, carapace, plastron, the four limbs and the tail (Fig. 2), just missing some claws of the right fore and hindlimbs. The specimen is on display at the Messel Pit exhibit, Senckenberg Museum of Natural History in Frankfurt. The most anteroventral region of the skull is not fully prepared and is still covered by rock matrix. Cervicals 1 to 4 are preserved and prepared, the rest of the cervicals are probably still inside the rock matrix. To avoid damaging the specimen by removing its stable exhibit frame, photos of the ventral surface of the skeleton were taken at an angle (see Figs. S1–S4); however, they suffice to analyze the morphology of some elements including the plastron, pectoral girdle and the ventral surface of the carapace.

#### Skull and lower jaw

The skull is volumetrically well preserved without evidence of crushing, and is missing only the most anterior portion of prefrontals (Fig. 3). On the ventral surface (Figs. 3A and 3B), the interorbital bar is narrow and formed by the prefrontals and frontals. Each orbital ring is made from a contribution of the prefrontal, frontal, postorbital, jugal, and maxilla. The prefrontals contact each other medially, the maxillae laterally, and the frontals posteriorly. The frontals contact each other medially, the postorbitals laterally, and the parietals posteriorly. The postorbitals form a very narrow bar between the orbits and the temporal fenestra and have a lateral contact with jugal. The parietals and frontals exhibit a sculpturing pattern similar to striations. The parietal table (most dorsal surface) is narrow due to the very advanced temporal emargination. The parietals contact the frontals anteriorly, the postorbitals anterolaterally, the prootics posterolaterally, and the supraoccipital posteriorly. The prootic and all other bones that contribute to the otic chamber are well exposed dorsally due to the very advanced temporal emargination, which has also a contribution from the jugal. The foramen stapediotemporale is restricted to the prootic, located very close to the sutural contact between this bone and the quadrate. The supraoccipital forms a very narrow ridge at the roof top of the skull, contacting the parietals anteriorly and the prootics and exoccipitals laterally and posteriorly and forming a long crista supraoccipitalis that ends in a rounded edge far beyond the level of squamosals. Small portions of the quadrates are exposed dorsally as part of the otic chamber. The squamosals form a very long and narrow posterior process; anteriorly they contact the quadratojugals, and medially the prootic and opisthotic. The most lateral portions of both corpora hyoidis are visible at the temporal fenestra, forming a convex margin. The cornu branchiale (CB I) of the hyoid apparatus is a narrow and very long bone. Only the posterior-most region of the ventral surface of the skull (basicranium) is prepared. It preserves most of the medial and posterior portions of corpus hyoidis, as well as dislocated pieces of corpus branchiale II (Fig. S2).

The left lateral view of the skull (Figs. 3C and 3D) shows that the temporal emargination was very shallow. The maxilla contacts the prefrontal dorsally and the jugal posteriorly. The quadratojugal is a narrow bar in anterior contact with the jugal and posterior contact with the quadrate and the squamosal. The cavum tympani is formed by the quadrate and covered dorsally by the squamosal, having a very narrow and deep incisura collumela auris. The antrum postoticum is extremely reduced. Also seen in lateral view, the lower jaw (left ramus) is well preserved, formed principally by the dentary, which exhibits a large foramen dentofaciale majus. It is in posterior contact with the surangular and coronoid. The surangular exhibits a small and circular foramen intermandibularis caudalis.

#### Shell

The carapace is complete (Figs. 2A and 2B), being almost circular in outline with its posterior edge straight. The sculpturing pattern consists of pits and ridges, the pits coalescing into grooves at the lateral regions of the carapace. The nuchal is slightly more than three times as wide as long, having a shallow notch medially. Neural series is composed of seven elements. Neurals 1–4 are hexagonal in shape and wider posteriorly (at the level of the intercostal junction). Neural 5 is almost rectangular. Neural 6 is hexagonal but in contrast to neurals 1–4, wider anteriorly. Neural 7 is small and almost pentagonal in shape. Eight pairs of costals are well preserved. Costals 1–6 are completely separated medially by the neurals, while costals 7 and 8 have a median contact, equal to half of the length for costals 7 and the full length for costals 8. Laterally, at the margin of costals, the most lateral tips of costal ribs are usually exposed, but they are short and terminate in a rounded margin. Peripherals are completely absent as well as the preneural and any evidence of scales.

The plastron is well preserved in ventral view (Fig. S3), but most of the entoplastron region is obscured by the attachment of the metalic frame for the exhibit-support of the specimen. The bridge length is very short and the epiplastron has a J-shape. The central region of the plastron has a very reduced level of ossification, so that the hyo-, hypo- and xiphiplastral bones lack of long and continuous medial contact between each pair, touching each other via processes. The most lateral end of both hyo- and hypoplastral bones are clearly visible from the dorsal view of the shell (Figs. 2A and 2B) ending in a pair of long and narrow horns (processus cardinus masculi anterior for hyoplastral bones and processus cardinus masculi posterior for hypoplastral bones).

#### Pectoral girdle, limbs, and tail

The left scapula, acromion process and the coracoid are well preserved (Fig. S4), the coracoid being the longest of the three elements and forming a slightly curved and distally wider bone. The four limbs are well preserved and with the major long bones articulated or slightly displaced from their original articulation points, the femur is slightly longer than the humerus (Fig. 2C). The left humerus preserves the ectepicondylar foramen, which runs openly along the anterodistal margin of the bone (Fig. 2E). The tail is very long and composed of at least fourteen caudal vertebrae with very pronounced lateral processes (Fig. 2D).

### SMF ME 1003

Nearly complete and fully articulated skeleton (Fig. 4) (63 cm maximum length and 49 cm maximum width as preserved), including the articulated skull and lower jaw, carapace, plastron, the neck, the four limbs, and the tail. Together with SMF ME 1211, it represents one of the most complete specimens for this taxon. Considering that it is well preserved ventrally, I focus principally on the ventral description of this specimen, noting that it shares all the dorsal features described for skull and shell of SMF ME 1211, as for example shape and number of carapace bones.

#### Skull and neck

The ventral surface of the skull is fully covered by the bony elements of the hyoid apparatus and the articulated lower jaw (Figs. 4A and 4B). The corpus hyoidis is flat and well ossified, ending posterolaterally in two processes. The cornu branchiale I is a narrow and very long posterodorsally projected bone. The left cornu branchiale II is particularly well preserved, being a wide and long bone, strongly ossified, and formed by the synostosis of at least two elements. The left squamosal is well preserved and exhibits the long and narrow posterior process and its ventral contact with the quadrate. The atlas and cervicals 2 and 3 are also well preserved. The atlantal centrum is small and square in shape, with a slightly concave posterior articular surface. Cervicals 2 and 3 are very long, almost three times as long as wide, exhibiting ventral keels that run along the entire centrum; pre- and postzygapophyses are very prominent and subelliptical in shape. The articulation between cervicals 2 and 3 is opisthocoelous.

#### Plastron, pectoral and pelvic girdles

The plastron is complete and articulated, with only the right xiphiplastron is slightly displaced from its original position (Figs. 4C and 4D). Both epiplastra have J-shaped and moderately projected anteriorly. The entoplastron is boomerang-shaped with an internal angle of approximately 100 degrees and a convex medial margin. The hyoplastra are almost rectangular in shape without marked shoulder and lack a mutual medial contact. They have a pair of processus cardinus masculi anterior, (single on the left side, double on the right side). The hypoplastra contact each other medially via a two processus hypoplastralis medialis anterior; both exhibit a well develop inguinal notch, a single rounded processus hypoplastralis medialis posterior, and a pair of processi cardinus masculi posterior. Both xiphiplastra are in short medial contact via a single dentate processus xiphiplastralis anterior, and in anterolateral contact with the hypoplastra.

Both scapulae with acromion processes are well preserved, as well as the most proximal portions of both coracoids. The dorsal processes of both scapulae are in medial contact, and the angle between the scapula and acromion process is slightly less than 90 degrees. The glenoid fossae are visible and show the sutural contact between the scapula and the coracoid. Of the pelvic girdle, the ischia are in medial contact and exhibit a narrow and well-developed metischial process, which ends in an acute tip.

### WDC-C-MG-311

Nearly complete articulated skeleton (42 cm maximum length and 35 cm maximum width as preserved) (Figs. 5 and 6). The neck region is completely covered by the skull. The ventral surface of the skeleton is still not fully prepared, having a significant amount of rock matrix, particularly at the lower jaw and neck region. As a result, it cannot be ascertained, for example, whether the fully cervical series is preserved.

#### Skull and lower jaw

Skull slightly affected by crushing, particularly at its right anterolateral margin and the posterior region. The lower jaw is articulated and slightly crushed too, especially the right ramus. On the dorsal surface of the skull (Figs. 6A and 6B), the parietals and frontals exhibit a sculpturing pattern similar to striations that run parallel to the length axis of the skull. The left prefontal is well exposed dorsally due to crushing, contacting the premaxilla, maxilla, and frontal and contributing to the anterior margin of the orbital ring. The frontals contact one another on the midline, the prefrontals anteriorly, and the postorbitals and parietals posterolaterally and contribute to the orbital ring laterally. The parietals contact one another on the midline, the postorbitals and prootics laterally, the frontals anteriorly, and the supraoccipital posterolaterally. The left postorbital contributes anteriorly to the orbital ring, having a lateral contact with the jugal and a medial contact with the frontal and parietal. The prootics and all other bones that contribute to the otic chamber are well exposed dorsally due to the very advanced temporal emargination. The foramen stapediotemporale seems to be restricted to the prootic and is located very close to the sutural contact between that bone and the quadrate. A small portion of the supraoccipital is exposed dorsally.

In lateral view of the skull (Figs. 6C and 6D), a small portion of the premaxilla is visible anteriorly. The maxilla contacts the prefrontal and premaxilla and the jugal posteriorly and dorsally contributes to the orbital ring. The cheek emargination region is affected by crushing and is partly covered by rock matrix. The quadrate is well preserved, covered dorsally by the squamosal, contacting the quadratojugal anteriorly, and ventrally in contact with the articular from the lower jaw. The left dentary is well exposed and forms most of the left ramus of the lower jaw. The bone contacts forming the coronoid region of the lower jaw are not well defined, but part of the coronoid, surangular, and articular are visible. Visible just under the left ramus of the lower jaw, due to crushing of the ventromedial surface, is the right ramus, but sutural contacts are not clear.

#### Shell

The carapace is complete (Figs. 5A and 5B), having an almost circular outline with slightly straight nuchal and pygal region margins. The dorsal sculpturing pattern is of pits and grooves (Fig. 5C), the latter being more distinct laterally and contributing to short ridge-like prominences. The nuchal is at least three times as wide as long and in posterior contact with costals 1 and neural 1. The neural series is composed of seven elements. Neural 1 is almost rectangular in shape and restricted to costals 1; neural 2 is small and restricted to costals 2. However this arrangement is due to slight crushing of this region of the carapace. Neural 3–6 are hexagonal in shape with narrow anterior ends, and neural 7 is pentagonal and restricted to costals 7. Costals 1–6 are completely separated medially by the neurals, while costals 7 and 8 are medially in contact. Laterally, at the margin of costals, the lateral ends of costal ribs is usually exposed, ending in a rounded tip.

In ventral view of the shell (Fig. 5D) most of the plastron is still covered by rock matrix, however portions of both J-shaped epiplastra are visible, as well as the most lateral portions of both hyo- and hypoplastra. The posterolateral-most tip of the right hyoplastron and the left hypoplastron ends in a pair of horns (processus cardinus masculi anterior and posterior, respectively).

#### Limbs and tail

The four limbs are articulated and nearly complete, preserving almost all their five digits, especially in the right forelimb (Figs. 5A and 5B). Digits I–IV are almost the same length. The tail is also complete and articulated, formed by at least fourteen caudal vertebrae. Part of the left scapula is exposed close to the left humerus, but its complete shape cannot be well defined because it is mostly covered by rock matrix.

### WDC-C-MG-310

Nearly complete skeleton with most of its bones completely disarticulated but kept inside the shell as a cradle (28 cm maximum length and 19 cm maximum width as preserved) (Fig. 7). Most of the limb long bones as well as some cervical vertebrae and pectoral girdle elements are preserved in the ventral cavity of the shell together with the plastron. Both the carapace and plastron suffered some degree of crushing, fracturing and displacement of some of the bones. The skull and some distal elements from a limb (potentially from the left forelimb) are preserved on the dorsal surface of the carapace.

#### Skull

The skull is relatively well preserved, displaced, and located on the left side of the carapace. On the dorsal surface (Figs. 8A and 8B), the snout and orbits are broken and slightly crushed, but portions of the prefrontals and frontals are preserved, and the ventromedial surface of the left maxilla is seen. The roof top of the skull is formed by the frontals and parietals exhibiting a bone surface with striations, and not affected by crushing, clearly showing the sutural contact between parietals, frontals, and postorbitals. The temporal emargination is highly advanced, completely exposing the otic chamber and the processus trochlearis oticum. The contact between the prootic and the quadrate is clearly marked on the left side of the skull, as well as the contact between the opisthotic and the supraoccipital. The left cavum tympani is completely collapsed, and the squamosal and condylar regions are too poorly preserved to clearly establish the sutural contacts between bones.

#### Shell and limb bones

The carapace is nearly complete, but highly affected by fracturing, displacement, and crushing (Figs. 7A and 7B). The dorsal surface of neurals, costals, and nuchal exhibit the pit-and-groove sculpturing pattern described above. The nuchal is wider than long and facing mostly anterolaterally as preserved. At least six neurals are clearly defined. Neural 1 reaches right costal 2, and neurals 2–5 are hexagonal in shape and wider posteriorly at the level of the intercostal suture. Seven pairs of costals are preserved, but most of them are broken and overlap one another.

In ventral view of the shell (Figs. 7C and 7D), the right hyo- and hypoplastra, although slightly displaced, are still in contact with each other, but they are broken into at least three big pieces. The lateral-most tips of the right hyoplastron and the left hypoplastron ends in pair of processus cardinus masculi anterior and posterior, respectively. Most of the long limb bones are preserved, but some of them are covered by others or by portions of the plastron principally in ventral view. A well-exposed right femur is clearly visible on the right portion of the shell, exhibiting a very deep concavity between major and minor trochanters. The left scapula with acromion process is located over the left hyoplastron, and the most proximal portion of the coracoid is also preserved.

### WDC-PBP-2015-DE001

Complete carapace, disarticulated portions of the plastron, some pelvic girdle elements including both pubes and both ischia, most of the left and right forelimb bones, some bones of the left hindlimb, as well as a cervical (8?) and two caudal vertebrae are preserved in a block rock, all of them belonging to the same individual (Figs. 9A and 9B).

#### Shell

The sculpturing pattern of the dorsal surface of both the carapace and plastron bones is made of pit-and-groove type, with smooth strips at their lateral margin, as described above. The nuchal bone is more than three times as wide as long, exhibiting a shallow medial notch. The neural series is composed of seven elements. Neurals 1–4 are hexagonal in shape and slightly wider posteriorly. Neural 5 is almost rectangular in shape. Neural 6 marks the reversal in the arrangement of neurals, being slightly wider anteriorly, in contrast to neurals 1–4. Neural 7 is the smallest of the series and is pentagonal in shape. The carapace has eight pairs of costals, with costals 7 and 8 having a median contact between each pair. The preserved plastral elements correspond to a portion of the left hypoplastron, a potential portion of a hyoplastron preserved in an almost vertical position, and both xiphiplastra, although these are disarticulated and exposed in dorsal surface.

#### Other postcranial elements

Both pubes are preserved, the left one covering the medial portion of the right one. The left pubis forms a curved plate anteromedially, while distally it has the strong process for the articulation with the ischium. Both ischia are preserved, having a narrow and well developed metischial process, ending in an acute tip. The left coracoid lamina is also preserved, exhibiting an oval-blade shape, with slight medial curvature.

Both humeri are preserved and their length is almost the same as for the femur. The femur has a very deep concavity between minor and major trochanters. Most of the small fore- and hindlimb elements are preserved in a disarticulated state, including some of the claws. One cervical vertebra, potentially cervical 8 is also preserved, exhibiting well developed and laterally projected prezygapophyses, and being small and square in shape. Four caudal vertebrae are preserved near the ischia, being almost square in shape, and at least in one of them the amphicoelous condition is clearly defined.

## Results and Discussion

### Taxonomy

Three of the five specimens described herein (SMF ME 1211, 1003 and P522) represent some of the most complete and best preserved Paleogene (Eocene) pan-trionychid turtles so far known in the entire world. All of them represent adult individuals and exhibit a very conservative morphology, having the same number of neurals and costals and the same shape and proportions of skull and postcranial bones, even though one of them is from a different locality (Geiseltal), which is slightly younger. This similarity supports their attribution to the same taxon, *Palaeoamyda messeliana* nov. comb., which was previously reported as abundant in Eocene localities of central Europe (Karl & Müller, 2008, references therein).

### Comparisons

**Figure 10:**
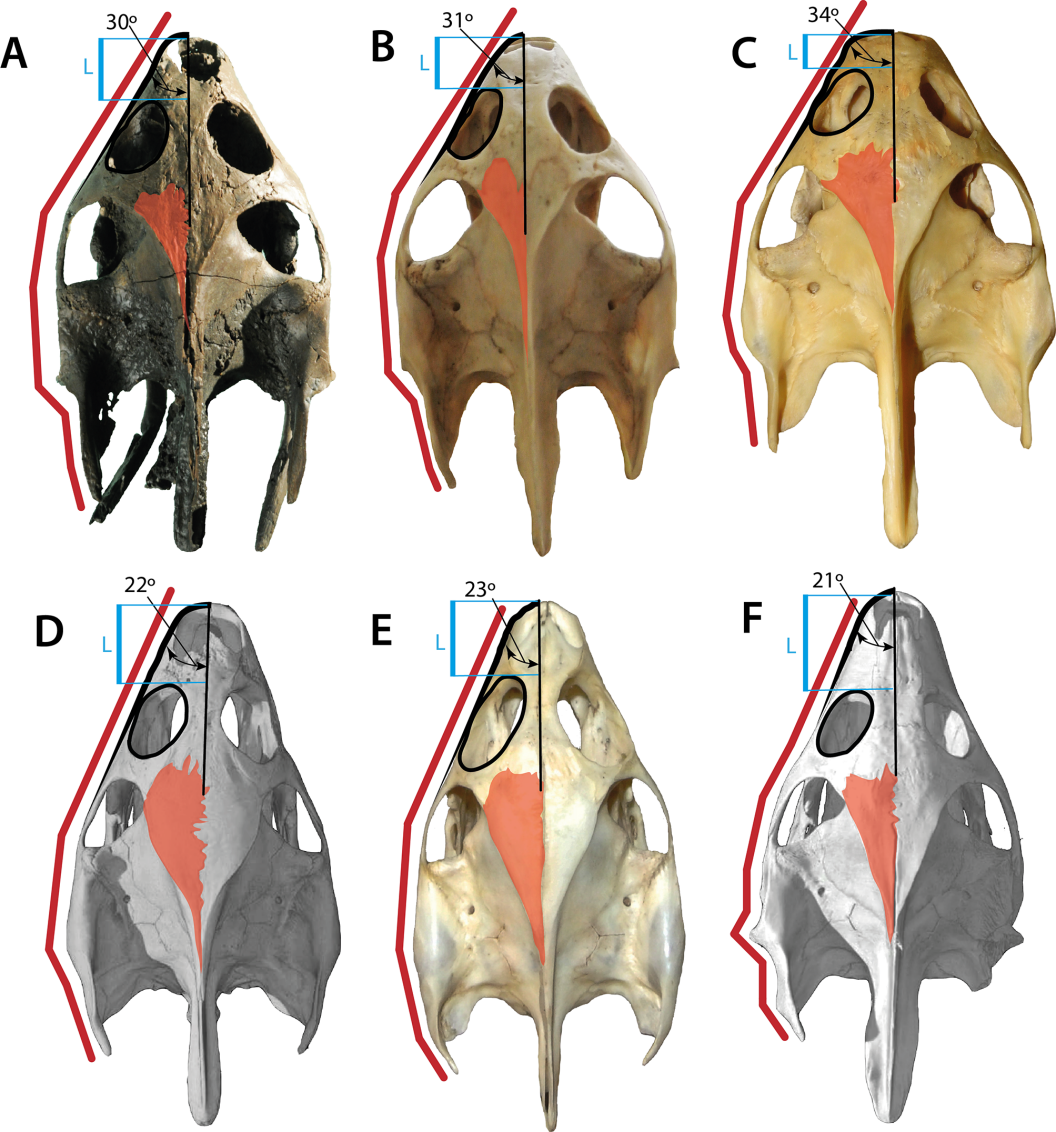
Skulls of some pan-trionychids in dorsal view. (A) *Palaeoamyda messeliana* SMF ME 1211; (B) *Amyda cartilaginea* NMW 32232. (C) *Rafetus euphraticus* NMW 1856. (D) *Apalone mutica* PCHP 2746. (E) *Apalone spinifera* NMW 1876. (F) *Trionyx triunguis* PCHP 4559. All skulls converted to almost the same length. Left lateral margin of the snout (green line), complete left lateral of the skull (red line), orbital ring (black line), dorsal roof top exposure of left parietal (pink area), and narrower region of the left portion of the interorbital bar (blue area).

The skull of *Palaeoamyda messeliana* resembles in many aspects the skull of the extant species *Amyda cartilaginea* (see Figs. 10A and 10B), both exhibiting a similar shape, proportions of the snout, which is slightly wider in the extant *Rafetus euphraticus* (Fig. 10C), the middle Eocene *Trionyx ikoviensis* (Danilov et al., 2011, Fig. 2) from Ukraine,and the early Eocene *T. michauxi* (Broin, 1977, Plc. XI) from France. Although, intraspecific variation can occur at the snout region, as it has been shown for *Apalone (Trionyx) ferox* by Dalrymple (1977), features as for example the angle between the lateral margin of the skull and its midline seems to be very distinct and conservative, for at least 8° average difference between the genera of two groups (*Palaeoamyda*, *Amyda*, *Rafetus*) versus (*Trionyx* and *Apalone*) (see Fig. 10), more specimens should be examined by future studies to support these observations. *Palaeoamyda messeliana* also shares with *A. cartilaginea* a very advanced temporal emargination reducing the area of exposure of parietals in the skull table; exposure is slightly greater in *T. ikoviensis*, *T. michauxi*, *Apalone mutica* (Fig. 10D), *Ap. spinifera* (Fig. 10E) and *T. triunguis* (Fig. 10F). The length between the most anterior margin of the orbit and the most anterior margin of the nasal fossa in dorsal view is intermediate in *P. messeliana* as in *A. cartilaginea*, in contrast to a shorter length exhibited by *R. euphraticus*, and much longer in *Ap. mutica, Ap. spinifera*, and *T. triunguis* specimens examined in this study. The shape of lateral outline of skull is also very similar between *P. messeliana* and *A. cartilaginea,* both sharing similar angle at the snout region (30° and 31° respectively), and similar lateral margin shape of the squamosal. The angle of the snout is slightly greater (between 34–38°) in *R. euphraticus, T. ikoviensis*, and *T. michauxi*, and much lesser (between 21–23°) in *Ap. mutica*, *Ap. spinifera,* and *T. triunguis* (see Fig. 10). However, intraspecific variations can occur in many of these features, including for example the shape of the crista supraoccipitalis, as it was demonstrated for *Ap. ferox* by Dalrymple (1977, Fig. 4). Having this into account, these morphological features should be treated with careful and avoid to use them in the diagnosis of genera or species pending of an extensive sampling and analysis of their intraspecific variation. However, they should be included in the discussion or comparisons as it is done here.

The sculpturing of the dorsal (carapace) and the ventral (plastron) bone surfaces exhibited by *Palaeoamyda messeliana* resembles the pit-and-groove pattern exhibited by many pan-trionychids, seen in all metaplastic portions (except at the most lateral margins of the nuchal and costals), grading into the pattern characteristic of the Paleocene members of *Hutchemys* from North America (Joyce et al., 2009). Additionally, the carapace of *P. messeliana* lateral ridge-like sculpturing pattern, something that was figured and considered as exclusive of the genus by Karl (1998, Fig. 2R); however, it is also present, for instance, in the middle Eocene *Trionyx ikoviensis* from Ukraine (Danilov et al., 2011, Fig. 4G). The degree of ossification of the carapace, particularly of the lateral portions of costals is similar to the extant members of *Amyda cartilaginea* NHMW 32232 and specimens figured in Karl (1997, Fig. 2), which is very advanced, such that only the lateral-most tip of the ribs are visible in dorsal view of the carapace, and with the complete absence of the posterior nuchal fontanelle. Reduced lateral ossification, extensively exposing the ribs laterally and producing a posterior nuchal fontanelle, is very common in members of *Apalone*, e.g., *Ap. spinifera* NHMW 1876 and that figured in Sheil (2003, Fig. 4D), as well as the *Ap. ferox* specimens figured in Karl (1997, Fig. 3). *Palaeoamyda messeliana* shares with almost all other pan-trionychids the occurrence of eight pairs of costals, whereas in members of *Apalone* the most common condition is seven pairs (Meylan, 1987). The number of neurals (seven) is constant in all complete specimens described in here, as well as the shift in neural orientation at neural 6, which is also the condition of *Rafetoides (Trionyx) henrici* (Karl, 1997, Fig. 26). Seven neurals is also a very common condition in members of *Apalone* and *Cyclanorbis*, but for the rest of Pan-Trionychidae the most common condition is to have eight or more neurals completely separating the costals or (in some cases) at least separating the most anteromedial region of costals 8. *Palaeoamyda messeliana* and *R. (Trionyx) henrici* exhibit a shallow notch at the anteromedial portion of nuchal bone, but it is much less pronounced that in *Hutchemys* (Joyce et al., 2009, Fig. 2). The most common condition in the rest of Pan-Trionychidae is to have an anterior nuchal margin straight to slightly convex.

The plastron of *Palaeoamyda messeliana* and *Rafetoides (Trionyx) henrici* see Karl (1997, Fig. 29) also resembles the plastron of *Amyda cartilaginea* (see Karl, 1997, Fig. 8A; Fig. S1) in the shape of the entoplastron and the hyo-, hypo- and xiphiplastra. The hyo- and hypoplastra each have a pair of processus cardinus masculi anterior and posterior respectively (occasionally a single one in one of the sides), whereas in *Apalone* the common condition is a single horn (processus cardinus masculi anterior) in both hyoplastra (see Meylan 1987, Fig. 8). *Palaeoamyda messeliana* differs from *A. cartilaginea* and other pan-trionychids in reducing the number of horns (processus hypoplastralis medialis anterior and posterior) to one. Another difference between *P. messeliana* and *A. cartilaginea* is the length of the epiplastra, which is longer in *A. cartilaginea*. *Palaeoamyda messeliana* is similar to other trionychids, e.g., *Ap. spinifera* (Sheil, 2003, Figs. 6 and 7) in the shape and proportion of the limb bones (e.g., femora slightly longer than humeri), the number and proportion of manus and pes digits and bones size, and the shape of the pectoral girdle elements. The ischia of *P. messeliana* share with *A. cartilaginea* and other members of Chitrini (sensu Meylan, 1987) a very distinct, narrow metischial process that ends in acute tip, which is less pronounced and shortened in other pan-trionychids (see Meylan 1987, Fig. 21).

### Phylogenetic position of *Palaeoamyda messeliana*

An initial hypothesis of the phylogenetic position of *Palaeoamyda messeliana* and *Rafetoides (Trionyx) henrici* within Pan-Trionychidae was presented by Karl (1997) in his doctoral thesis (cladogram 3). He suggested that they form a monophyletic group outside the clade formed by Aspideretini and Trionychini (sensu Meylan, 1987, Fig. 32), with the exclusion of *Trionyx triunguis* from Trionychini. However, this hypothesis was a hand-generated tree, not the result of a repeatable, computer-based analysis using an explicit optimality criterion.

**Figure 11:**
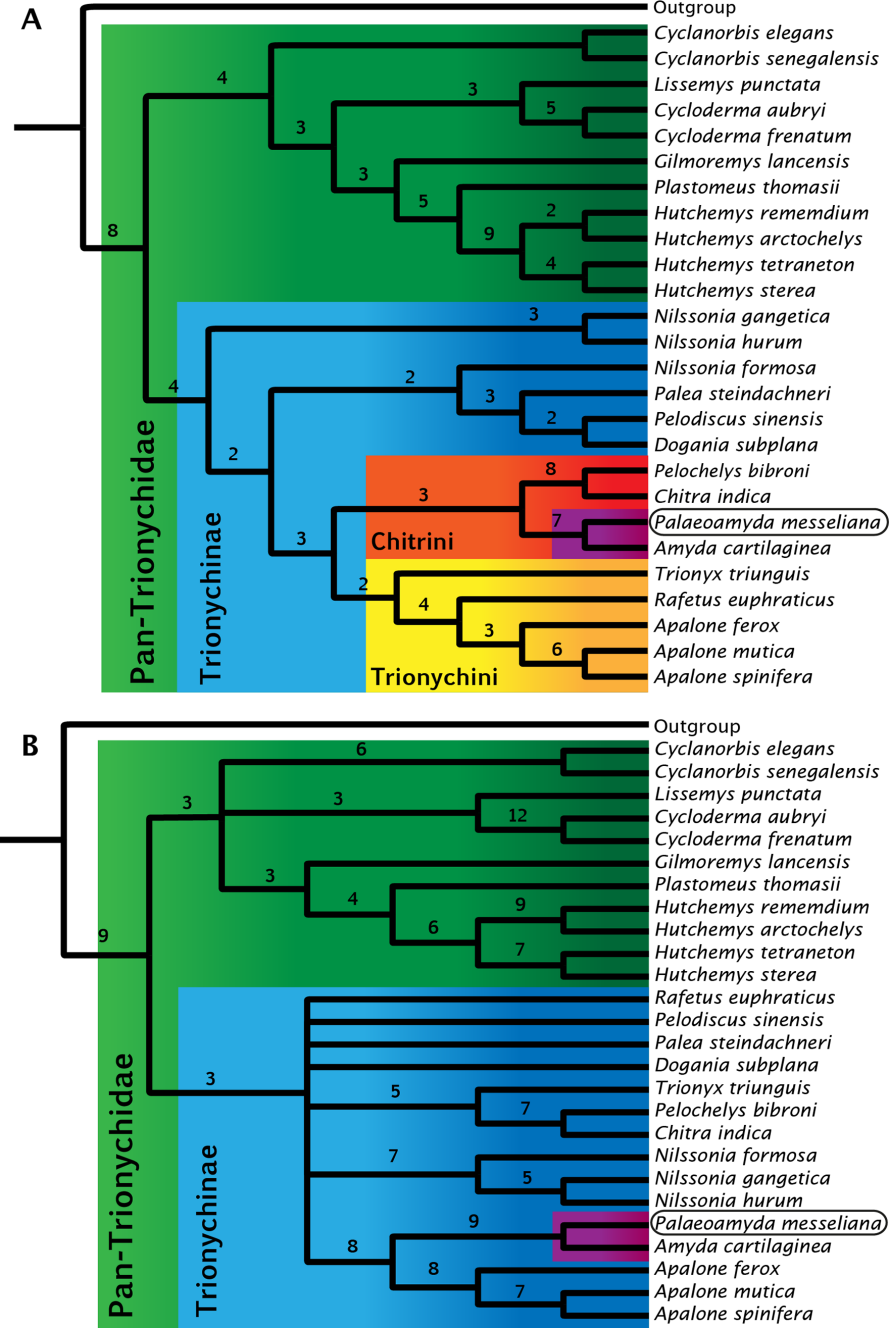
Strict consensus trees for Pan-Trionychidae (this study). (A) morphological data without enforced constraint topology, single most parsimonious tree (MPT), tree length = 261, consistency index = 0.471, and retention index = 0.623. (B) strict consensus from 16 MPTs, morphological data with molecular constraint topology enforced, tree length = 274, consistency index = 0.449, and retention index = 0.587. Bremer support index shown for each clade. Clades by color: Pan-Trionychidae (green), Trionychinae (blue), Trionychini (yellow), Chitrini (red), and *Amyda*(purple) this last including *Palaeoamyda messeliana*.

For the study of the phylogenetic relationships of *Palaeoamyda messeliana*, I incorporated it into the most recent character-taxon matrix of Joyce & Lyson (2011). Analysis of the matrix without using a molecular topological constraint for the extant taxa resulted in a single most-parsimonious tree (Fig. 11A) (Tree length = 261, Consistency index = 0.471, and Retention index = 0.623). In this tree *Palaeoamyda messeliana* is inferred to be the sister taxon of *Amyda cartilaginea*, forming a clade that is sister to *Pelochelys bibroni* and *Chitra indica*. The topology of Chitrini with regard to extant species is the same as that found by Meylan (1987, Fig. 32). Additionally, the topology obtained here resembles the one obtained by Meylan (1987) in the composition of Trionychini, including *Trionyx triunguis*. However, differences appear in the composition of Aspideretini and Pelodiscini. For example, *Nilssonia formosa* is found inside Pelodiscini here, whereas Meylan (1987) found it to be in Aspideretini. Also, inside Pelodiscini, *Pelodiscus sinensis* is found as sister taxon of *Dogania subplana.* The close relationship found here between *P. messeliana* and *A. cartilaginea* supports the hypothesis proposed by Karl (1998) and Karl (1999) that the European Paleogene pan-trionychids are part of a migration arriving from Asia.

The second phylogenetic analysis using the topological constraint based on molecular data (see ‘Material and methods’ section) resulted in sixteen most-parsimonious trees, strict consensus tree is shown in Fig. 11A, (Tree length = 274, Consistency index = 0.449, and Retention index = 0.587). *Palaeoamyda messeliana* and *Amyda cartilaginea* are again found as monophyletic clade, however this time closer related to *Apalone* spp. clade with high boostrap values supporting their close relationship.

Finally, I want to point out that an exhaustive revision and update of the characters and their states from the original Meylan (1987) character-taxon matrix must still be accomplished, which was out of the scope of this study. In particular, a new revision should include characters derived from detailed observations of CT scanned specimens as well as fixing some issues of redundancy in character definitions and states, that is for example the case of character 14 (16 of Meylan, 1987) “pleurals (costals) which meet at midline” which has the following states: 1. eighth only; 2. seventh and eighth or eighth only; 3. sixth, seventh, and eighth or seventh and eighth; 4. more than sixth, seventh, and eighth. The condition “eighth only” is repeated in two different states creating redundancy and subjectivity for the coding process. Additionally, in most of the cases the midline contact is partial for some of the costals, condition that should be also clearly defined in the states. Improving the characters definition and increasing the exploration of intraspecific morphological variations among extant species will potentially bring more stability to the phylogeny of Pan-Trionychidae, reducing the discrepancies between topologies obtained by morphology only data versus molecular information.

##  Supplemental Information

10.7717/peerj.2647/supp-1 Supplemental Information 1 Supplemental Information 1 Nexus file with the character-taxon matrix.

10.7717/peerj.2647/supp-2 Supplemental Information 2 Supplemental Information 2 Figures and photos of *Palaeoamyda messeliana* SMF ME 1211. **Figure 1**, View of the specimen located at one of the walls of the Messel Pit exhibit, Senkencberg Museum Natural History, Frankfurt, Germany. **Figure 2**, posterior region of the skull-lower jaw of *P. messeliana* SMF ME 1211. **Abbreviations**: bo, basioccipital; cbI, cornu branchiale I; cbII, cornu branchiale II; chy, corpus hyoidis. **Figure 3,** ventral view of the skeleton of *P. messeliana* SMF ME 1211, showing the plastral regions, and metallic frame that attach it to the wall of the exhibit. **Figure 4,** right pectoral girdle elements of *P. messeliana* SMF ME 1211.

10.7717/peerj.2647/supp-3 Supplemental Information 3 Supplemental Information 3 Plastron in dorsal view of *Amyda cartilaginea* NMW 32232.

## Institutional abbreviations

NHMW: Natural History Museum Vienna, Austria
PCHP: Chelonian Research Institute, Oviedo, FL, United States of America
SMF: Senckenberg Museum collections, Frankfurt, Germany
WDC: Wyoming Dinosaur Center, Thermopolis, WY, United States of America

## Additional Information and Declarations

### Data Availability

The following information was supplied regarding data availability: The raw data has been supplied as a Supplementary File.

### New Species Registration

The following information was supplied regarding the registration of a newly described species: Publication LSID: urn:lsid:zoobank.org:pub:A662842B-28A5-4A17-AC91-A9EDE8A75E79; Paleoamyda messelianus nov. comb. LSID urn:lsid:zoobank.org:act:4D55C528-72BF-40DC-8132-925C3C37B543.
