# Supplementary material for: Palaeoamyda messeliana nov. comb. (Testudines, Pan-Trionychidae) from the Eocene Messel Pit and Geiseltal localities, Germany, taxonomic and phylogenetic insights
Source: PeerJ. 2016 Oct 27;4:e2647. doi: 10.7717/peerj.2647 (PMC5088588; doi:10.7717/peerj.2647)

Figure 1

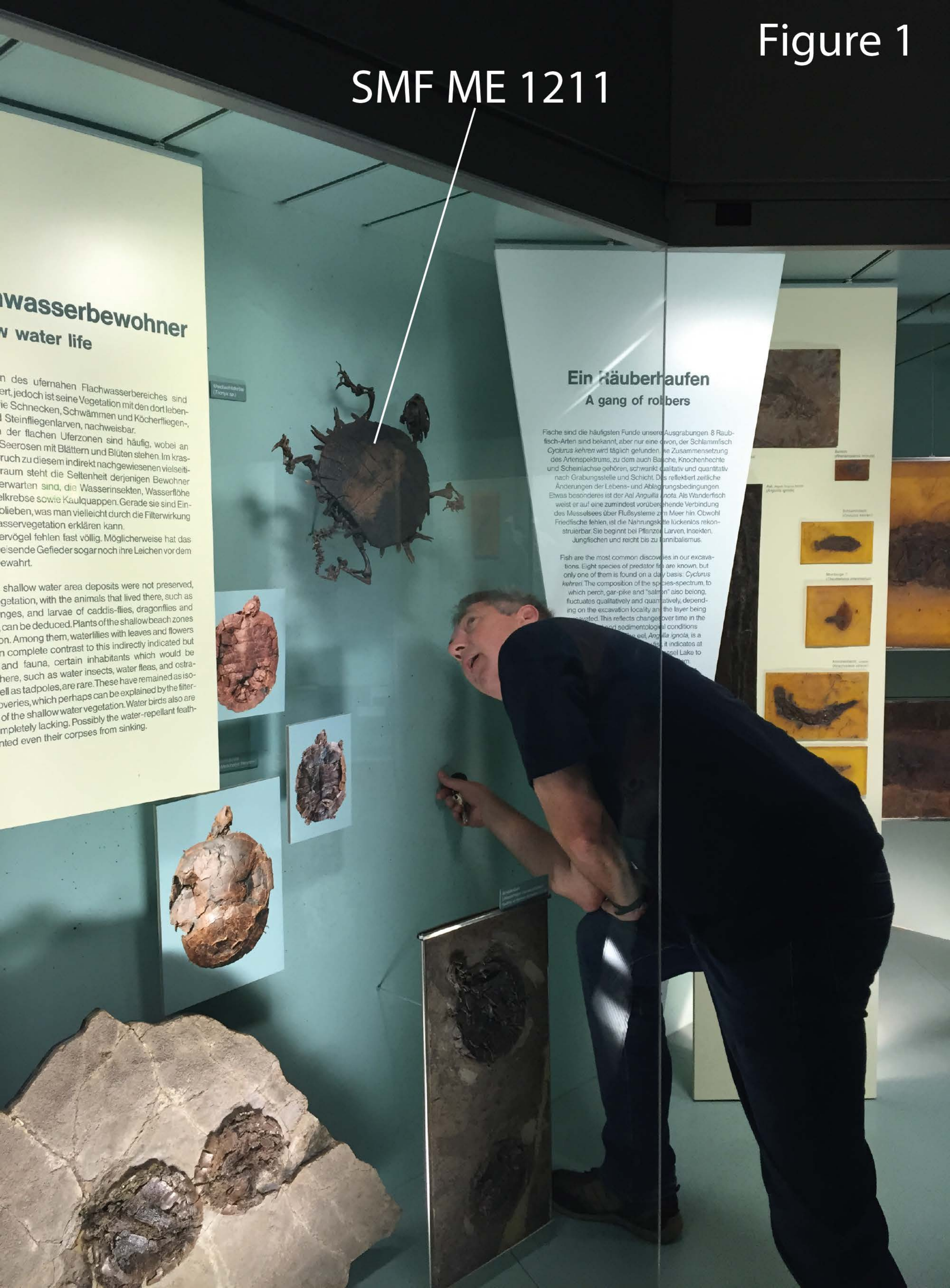

Figure 2

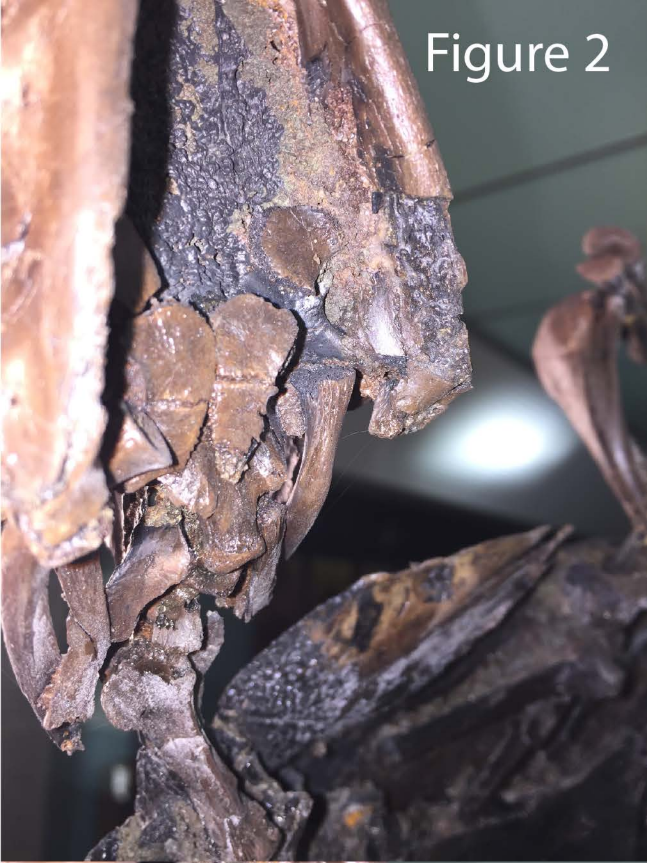

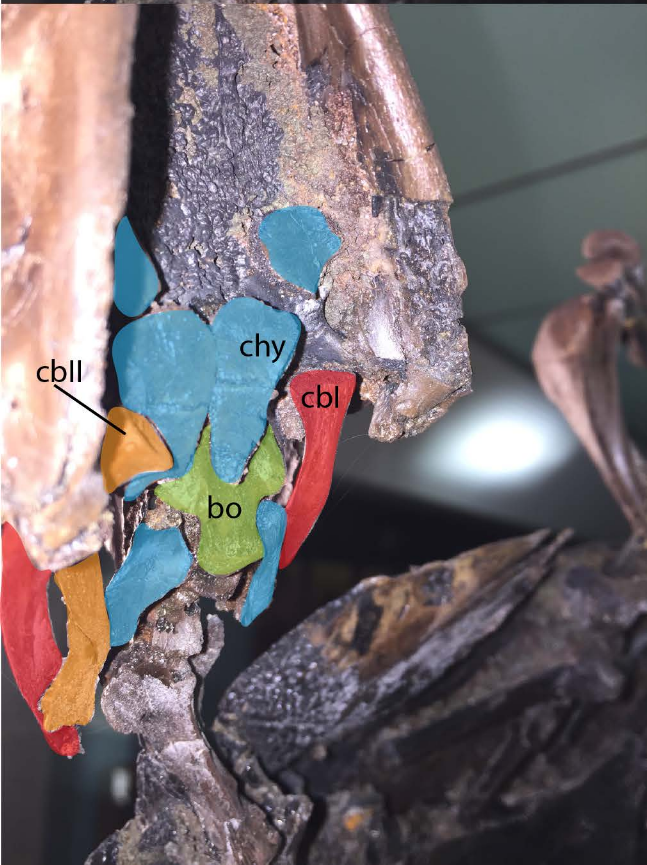

Figure 3

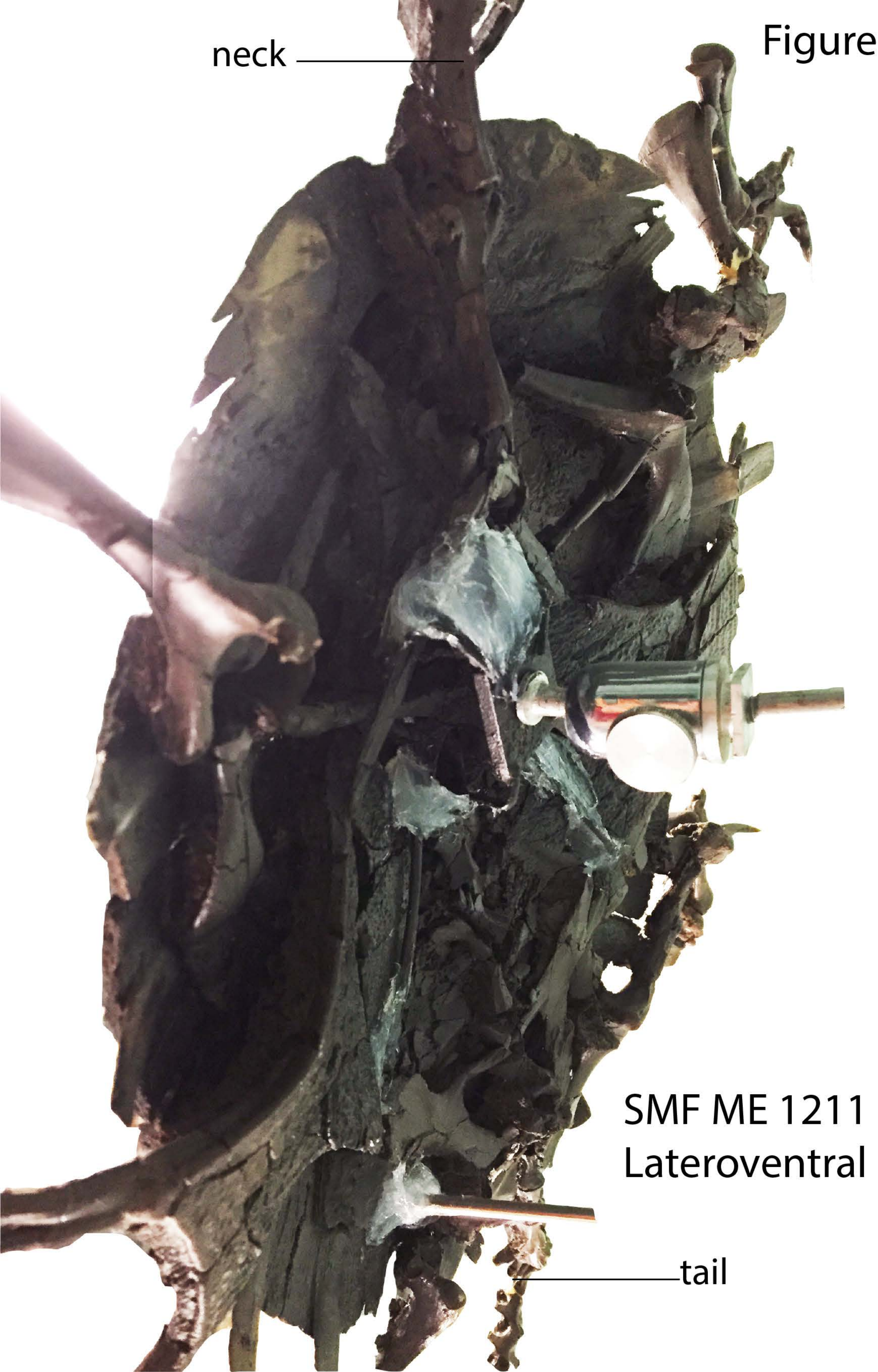

Figure 4

SMF ME 1211

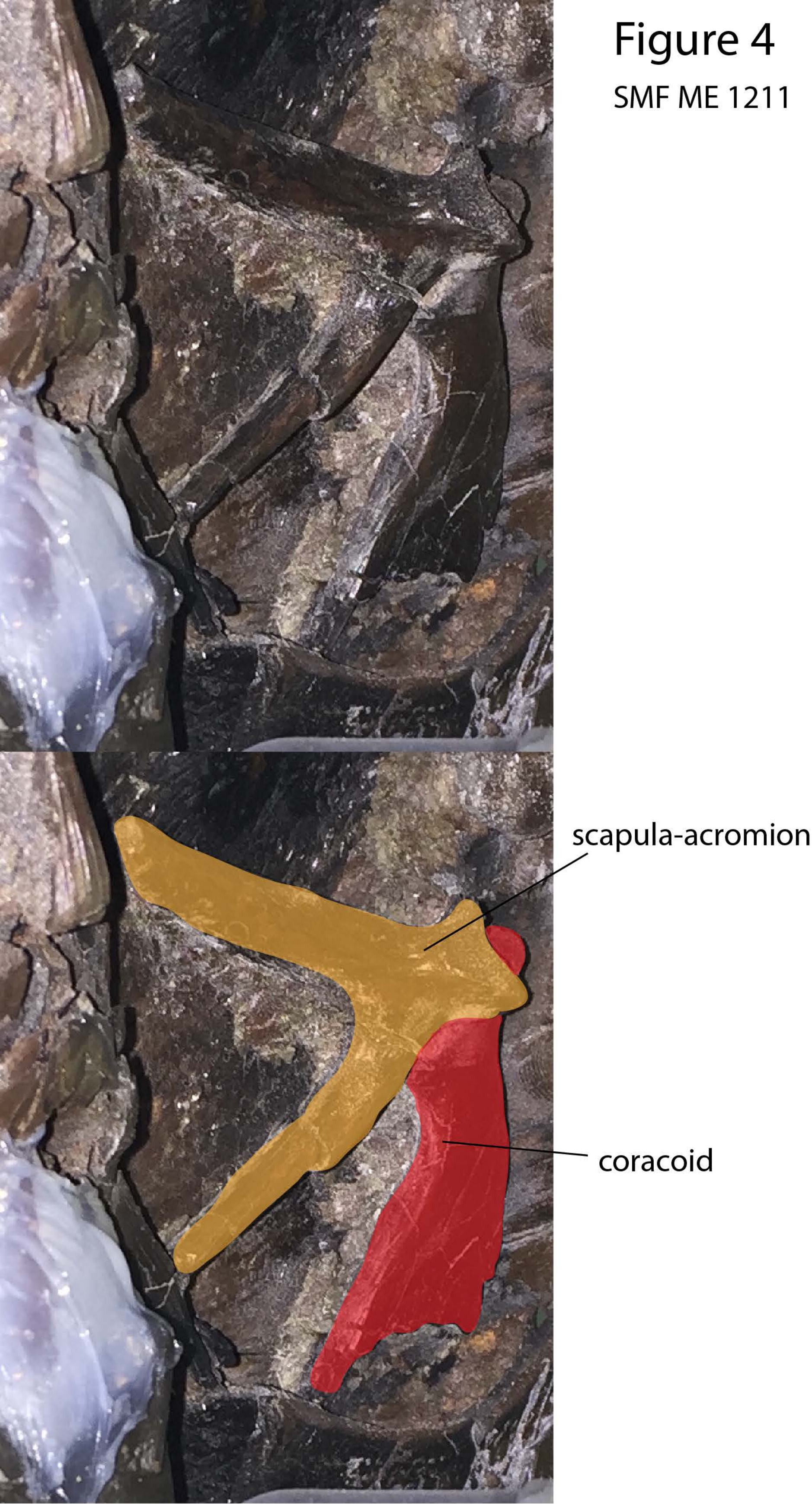

Supplement: Supplemental Information 2 — Figures and photos of Palaeoamyda messeliana SMF ME 1211. Figure 1, View of the specimen located at one of the walls of the Messel Pit exhibit, Senkencberg Museum Natural History, Frankfurt, Germany. Figure 2, posterior region of the skull-lower jaw of P. messeliana SMF ME 1211. Abbreviations: bo, basioccipital; cbI, cornu branchiale I; cbII, cornu branchiale II; chy, corpus hyoidis. Figure 3, ventral view of the skeleton of P. messeliana SMF ME 1211, showing the plastral regions, and metallic frame that attach it to the wall of the exhibit. Figure 4, right pectoral girdle elements of P. messeliana SMF ME 1211. [file peerj-04-2647-s002.pdf]
