# Supplementary figures and images for: Palaeoamyda messeliana nov. comb. (Testudines, Pan-Trionychidae) from the Eocene Messel Pit and Geiseltal localities, Germany, taxonomic and phylogenetic insights
Source: PeerJ. 2016 Oct 27;4:e2647. doi: 10.7717/peerj.2647 (PMC5088588; doi:10.7717/peerj.2647)

Figure 1

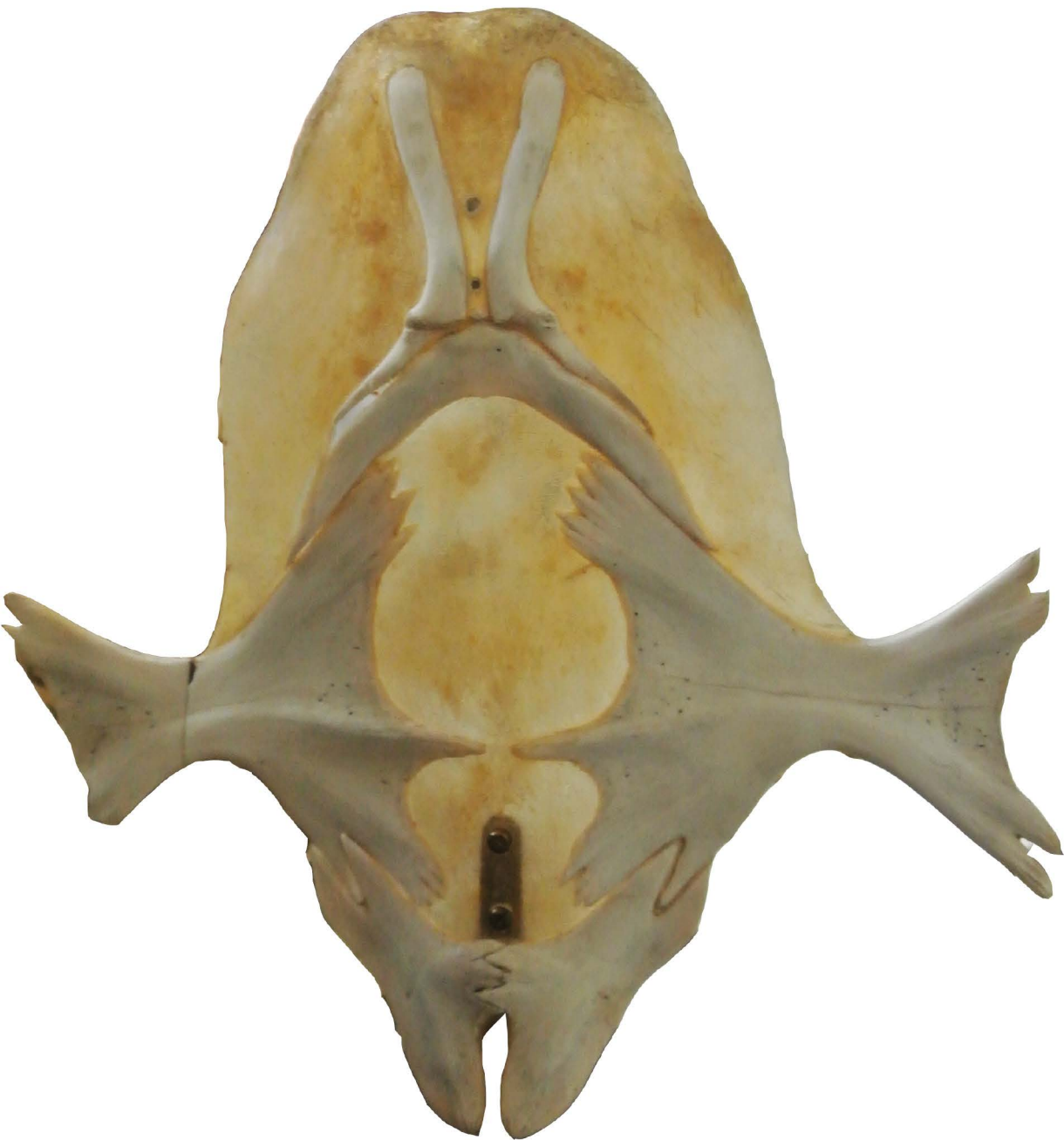

Plastron (vista dorsal) -  
*Amyda cartilaginea* NMW 32232

Supplement: Supplemental Information 3 — Plastron in dorsal view of Amyda cartilaginea NMW 32232. [file peerj-04-2647-s003.pdf]
